# Editorial: When the Shape Does Matter: Three-Dimensional *In Vitro* Models of Epithelial Barriers

**DOI:** 10.3389/fbioe.2020.617361

**Published:** 2020-12-01

**Authors:** Vanesa Fernández-Majada, María García-Díaz, Núria Torras, Michael Raghunath, Elena Martínez

**Affiliations:** ^1^Biomimetic Systems for Cell Engineering, Institute for Bioengineering of Catalonia, The Barcelona Institute of Science and Technology, Barcelona, Spain; ^2^Center for Cell Biology and Tissue Engineering, Institute for Chemistry and Biotechnology, Zurich University of Applied Sciences, Wädenswil, Switzerland; ^3^Centro de Investigación Biomédica en Red en Bioingeniería, Biomateriales y Nanomedicina, Madrid, Spain; ^4^Department of Electronics and Biomedical Engineering, University of Barcelona, Barcelona, Spain

**Keywords:** 3D cell culture models, organ-on-a chip, microengineered tissues, epithelial barriers, computer modeling

The Research Topic presented here includes a collection of selected pre-reviewed manuscripts that describe relevant recent advances in the fields of bioengineering and biophysics with a focus on the generation and analysis of novel three-dimensional (3D) *in vitro* models of epithelial barriers.

Recapitulating the 3D *in vivo* tissue environment at the biochemical, mechanical, and cellular level to obtain more physiologically relevant *in vitro* organotypic models is currently a very active line of research due to its direct impact on basic biology, drug testing, and disease modeling studies. Recent advances in cell biology and bioengineering techniques have opened new landscapes in the field. For example, it has been shown that the use of both artificial and natural matrices favors cellular self-organization in *in vivo*-like 3D tissue structures through cell-to-matrix signaling. This is the case for many engineered tissues, the liver among them. Although the was previously great success in reproducing the hepatic function *in vitro*, a method to include the biliary tree that is capable of removing toxic metabolites has not yet been developed yet. Funfak et al. demonstrate that normal rat cholangiocytes grown in 3D matrices based on artificial polymers (PEG) can form polarized and functional cysts comparable to those generated in natural polymers (Matrigel). In addition, the authors discovered that the elastic modulus of the substrate and the integrin ligand density directly affects cholangiocytes cyst formation frequency, size, and polarization. This work proves the suitability of artificial polymers for cholangiocytes organotypic models and decouples the biochemical and biophysical effects derived from the substrate on hepatic organoid formation.

The manuscripts of Macedo et al. and Darling et al. highlight the importance of upgrading standard 2D monocultures to more physiologically relevant *in vitro* models which represent not only the cell-to-matrix interface but also the interactions between the different cell types present in the *in vivo* organ. Both works focus on generating intestinal epithelial models. Macedo et al. engineered a 3D mucosal intestinal model comprising a collagen layer with embedded human intestinal fibroblasts, mimicking the cellular components and physico-chemical properties of the intestinal lamina propria and, on top, an epithelial monolayer composed of enterocyte-like (Caco-2) and mucus secretory-like (HT29-MTX) cells. They demonstrate that this advanced intestinal model better reproduces the cellular and physiological characteristics of the *in vivo* organ as proved by a more physiological absorption than that observed with standard 2D Caco-2 monocultures. Employing a similar experimental set up, Darling et al. investigated the effect of either the paracrine signals or the physical presence of fibroblast on the above growing epithelium. They show that when co-cultured in 3D and close to fibroblasts, Caco-2 monolayers display morphological characteristics such as enhanced columnar shape polarization and altered lateral membrane morphology like the *in vivo* intestinal epithelial monolayer. This improved cellular architecture translated into more physiological TEER and permeability values than those achieved with standard monocultures or with only the fibroblast-derived condition medium.

In addition to fibroblasts, basic intestinal epithelial cultures have been upgraded by incorporating the other cell types present in the *in vivo* tissue including endothelial, immune, and neuronal cells, their interface with the microbiome, as well as physical cues such as peristaltic movements. Steinway et al. extensively review recent progress in developing advanced micro-physiological intestinal systems and their use in evaluating normal intestinal physiology such as nutrient absorption, digestion, and secretion as well as metabolism, intestinal infection, inflammation, and cancer.

One of the most relevant applications of advanced *in vitro* organotypic models is the replacement of animal testing in the safety evaluation for consumer products and drugs. For instance, assessing the skin irritation potential of topical drugs and cosmetics by employing advanced *in vitro* cultures is already mandatory by law in the European Union since 2013. Wei et al. established an integrated organotypic skin model in a high throughput screening platform that enables large-scale dermal toxicology testing based on tissue viability, barrier integrity, and cytokine secretion. They employed bioprinting technology to produce organotypic skin constructs with controlled spatial cell layering, consistent cellular composition and distribution, and extracellular matrix (ECM) organization. With the aim of predicting oral drug administration-induced toxicity, De Gregorio et al. designed a microfluidic intestine-liver-on-chip (InLiver-OC). In contrast to single-organ systems, multiple organ-on-chip models recapitulate the complex *in vivo* intercellular and interorgan interfaces and therefore are excellent *in vitro* platforms to reliably model the enterohepatic circulation and first-pass metabolism, which are critical for assessing drug and xenobiotic metabolism. These authors employed a bottom-up tissue engineering strategy to build-up a physiologically functional 3D intestinal model with independent access to the apical and basolateral site of the tissue. This intestinal chamber is interconnected via microfluidic channels with a 3D Liver microtissue (HepG2-μTPs). With this advanced InLiver-OC, the authors thoroughly evaluated the intestine-liver metabolic and absorptive crosstalk of harmful model substances, such as ethanol. They propose their device as a tool to reduce the number of drug candidates and accelerate the pre-clinical screening process reducing animal testing.

Impedance spectroscopy (IS) is a promising technology, compatible with high throughput screening, that carries out a wide range of measurements in real time. It is been widely used as an analytical method of various physiological parameters, employing 3D tissue models and organ-on-a-chip devices. The applications, advantages, and limitations of IS as an analytical tool for advance culture systems are thoroughly discussed and reviewed by Gerasimenko et al..

Mimicking *in vitro* the complex 3D architecture of native tissues is important since it has been shown that it can better recapitulate the cellular and physiological functional properties of the *in vivo* organs. These complex 3D shapes of epithelial tissues require new analytical methods, both *in silico* and *in vitro*. Ioannou et al. present a 3D hybrid computer model that permits them to model epithelial movements that do not occur as planar deformations but which are, rather, characterized by out-of-plane mechanics and 3D effects such as tissue bending, invagination, and extrusion. The developed model simulates differential contractilities at the apical, basal, and lateral sides of the monolayer. On the practical side, Altay et al. introduce a new technology that allows for the histological processing of microstructured 3D intestinal models, preserving the fragile intestinal villi-like topology of the scaffolds. The high-resolution imaging of these 3D constructs enables a thorough study of cellular behavior on physiologically relevant matrix shapes. The authors reveal the positive effect of the substrate curvature on the cell shape and orientation along the villus axis and advocate for this as the cause of the more physiologically relevant barrier properties of the engineered intestinal tissues when compared with regular 2D monolayers.

This collection of original articles and reviews provides insights into the relevance of generating and properly analyzing micro-physiological *in vitro* epithelial tissues that aim to bridge the gap between traditional *in vitro* tools and costly animal studies.

## Author Contributions

EM and MR coordinated the research topic. VF-M coordinated the editorial and together with EM, MG-D, and NT wrote the manuscript. All authors contributed to the article and approved the submitted version.

## Conflict of Interest

The authors declare that the research was conducted in the absence of any commercial or financial relationships that could be construed as a potential conflict of interest.

